# Rural South Africans’ rehabilitation experiences: Case studies from the Northern Cape Province

**DOI:** 10.4102/sajp.v72i1.298

**Published:** 2016-09-16

**Authors:** Surona Visagie, Leslie Swartz

**Affiliations:** 1 Centre for Rehabilitation Studies, Stellenbosch University, South Africa; 2 Department of Psychology; Stellenbosch University, South Africa

## Abstract

**Background:**

Rehabilitation is often challenging in South Africa, but South Africans living in remote rural settings might experience unique challenges.

**Objective:**

This article interrogates issues of access to rehabilitation in a selected sample from rural South Africa through case studies.

**Method:**

This qualitative study utilised a case study design. Eight case studies were done in a purposively sampled rural town in the Northern Cape Province. Data were collected through in-depth interviews. Data were analysed according to the principles of interpretative phenomenological analysis.

**Results:**

The case study participants were not integrated into the community. They experienced higher levels of disability than one would expect from their impairments. Their impairments were not modified. No retraining of function was implemented. Early intervention and childhood development activities were not provided. Participants were not linked with self-help or peer support groups. Provision of assistive devices was challenged. Environmental barriers aggravated the situation.

**Conclusion:**

We theorise that one-on-one therapy is not the solution to the rehabilitation needs of persons with disabilities in remote, rural settings. We recommend a move to community-based rehabilitation and transdisciplinary teamwork supported by family members, community health workers and peer mentors. Therapists are ideally situated to explore the feasibility of such programmes and to pilot them in various communities.

## Introduction

As an essential component of healthcare services, rehabilitation provides a link between curative services and restoring the ability to enter or re-enter all facets of life (Department of Health [DoH] 2013; World Health Organization [WHO] 2011). To achieve this goal, rehabilitation should focus on modification of the impairment, compensation for loss of function and modification of the environment (WHO 2011).

Article 26 of the United Nations Convention on the Rights of Persons with Disabilities (UNCRPD), states that all persons with disabilities should have access to rehabilitation (UN 2006). Having ratified the UNCRPD, the South African government committed itself to ensuring that rehabilitation is available in urban and rural areas, is provided by multiple disciplines, starts as early as possible and focusses on participation and community integration (UN 2006). National policy dictates that rehabilitation be provided through the district health system according to the principles of primary healthcare, along a continuum from primary care through to tertiary and specialist services (DoH 2013; RSA 2012).

Traditionally in South Africa, rehabilitation has been provided on a one-on-one basis by individual service providers or multidisciplinary teams. A lack of South African evidence on the use of alternative options such as transdisciplinary teamwork, peer counselling and support, as well as utilising community-based health workers might mean that these options are seldom utilised, or if utilised not studied. Community-based rehabilitation workers have been trained and employed in large parts of the country. Chappell and Johannsmeier (2009) have found that in South Africa, as in Zimbabwe and Botswana, community-based rehabilitation (CBR) workers provided education and counselling and facilitated peer groups. Their services had a positive impact on activities of daily living, mobility and community integration.

Rehabilitation services in South Africa are challenged by a shortage of service providers and other resources (Bateman 2012; Maart & Jelsma 2013; Ntamo, Buso & Longo-Mbenza 2013), insufficient budgets and transport, short length of stay in acute hospitals, breakdown in communication, challenges with referral to secondary and tertiary services (Bateman 2012; DoH 2013), a medical model of service delivery, vertical programmes, poor intersectoral collaboration (DoH 2013) and language barriers (Maart & Jelsma 2013). Consequently, many South Africans do not attain and maintain ‘maximum independence, full physical, mental, social and vocational ability, and full inclusion and participation in all aspects of life’ (UN 2006:19). South Africans in remote rural areas may experience particular challenges regarding access to rehabilitation.

In this article we aim to interrogate issues of access to rehabilitation in remote rural settings through two cases drawn from a larger study on disability and healthcare access in rural South Africa. The International Classification of Functioning, Disability and Health is used as study framework (WHO 2001).

## Methodology

This qualitative study utilised a case study design, because case studies produce detailed, nuanced information on contemporary phenomena in a specific context. This information can facilitate a deeper understanding of complex societal issues and assist with the development of hypotheses that can inform action (Yin 2013).

The study was conducted in a rural town in the Northern Cape Province of South Africa. The town was purposively sampled as a study site because as a small, geographically isolated town in a semi-arid rural area, dependent on livestock farming, with low population density, poor infrastructure, high levels of poverty and inequities between various social groups, it is similar to many other rural towns in South Africa (Atkinson 2007). Healthcare and rehabilitation services were provided to the community through a nurse-driven, primary care service at a community healthcare centre (CHCC) with overnight facilities for up to five persons. Medical doctors, physio-, occupational- and speech therapists provided a weekly outreach service (4 hours per professional group per week) from a district hospital 200 km away. Secondary and tertiary healthcare was provided in larger centres up to 1000 km away. Therapists provided one-on-one treatment at the CHCC.

In the context of the larger study, 283 individuals who were likely to have a disability were identified in the study community (for methods, see Eide et al. 2015). These 283 individuals were considered the population for the current study. Because qualitative studies focus on depth of data rather than numbers of informants, eight case study participants were identified through purposive sampling (Yin 2013). Demographic information from the larger survey, prior knowledge of the population and information from field workers were used to select a heterogeneous group of participants with variation with regard to variables that might lead to different experiences on the issues under study. For the purposes of this article, we report on detailed case material collected from two of the eight case study participants. We selected these two because they provide the richest detail on the topic under discussion: provision of rehabilitation services to people with physical disabilities. The first case study explored the situation of 5-year-old Brenda, through three interviews with her grandmother and one with her mother. The second case study explored the experiences of Carel, an adult, through one interview each with him, his mother and his sister. Additional information on rehabilitation service delivery was gathered during a focus group discussion, with the three therapists providing outreach services to the community, in an effort to triangulate findings between service users and providers.

Data were collected in March and April 2012 by the primary author, who also transcribed and analysed it. All interviews were in Afrikaans, the first language of all the study participants. An interview guide, developed by the primary author, was used to ensure that all relevant aspects were covered during the interviews. Healthcare and rehabilitation were explored with a broad question asking participants about their needs and experiences in this regard. Further probing was based on their answers. The style of interviewing was that of a free-flowing conversation, and the thoughts and opinions of the participants were explored as they were presented, as recommended for qualitative research (Silverman 2013).

Data were analysed according to the principles of interpretative phenomenological analysis (IPA) (Smith, Flowers & Larkin 2009). IPA aims to comprehend phenomena from the perspective of the person experiencing them in order to enhance understanding of the phenomenon. However, it is never possible to completely understand the experience of another; thus in IPA the researcher tries to create a coherent third-person account of the participant’s experience and make sense of another’s world through interpretation (Smith et al. 2009).

In accordance with IPA (Smith et al. 2009) and case study theory (Yin 2013), each case was analysed separately before cross-case analysis was done. Transcribed interviews were printed on the middle third of a page. During multiple readings, explanatory notes and comments on the data were penned in the left-hand column. Following this the right-hand column was used for noting emergent themes. Finally, connections between themes were identified and similar themes were clustered together (Smith et al. 2009) under superordinate themes (a theme or concept that encompasses the themes clustered under it), of which rehabilitation, the focus of this article, was one. The other superordinate themes were as follows:

- the conceptualisation of disability
- healthcare systems used by participants
- barriers and facilitators to healthcare access (Visagie & Schneider 2014)
- rehabilitation
- vulnerability.

The credibility, applicability, consistency and neutrality of the study were addressed through prolonged engagement, triangulation of sources, thick description, producing a text that would allow readers to share in the lived experiences of the study participants, and reflection (Silverman 2013; Tracy 2010; Yin 2013).

### Ethical considerations

The study was registered with the Health Research Ethics Committee at the University of Stellenbosch, and written permission was obtained from the Northern Cape Department of Health. Study participation was voluntary. No data were collected before informed consent had been obtained in writing from participants or their legal guardians. Permission to digitally record interviews was also obtained. We treated all information and the identity of participants with the strictest confidence. A pseudonym was used during the analysis and dissemination of data. However, data were collected in a small town and there is a possibility of readers identifying the participants from the descriptions (Tracy 2010). Thus, we have presented data in a manner that would not be hurtful or damaging to participants should they be identified.

## Results

### Brenda

Brenda^1^ was born through an emergency caesarean section when her mother was 7 months pregnant. Her mother, 16 years old at the time, suffered from pre-eclampsia. Diagnosed with Cornelia de Lange syndrome, Brenda showed the typical physical features, such as small stature and upper limb deformities (she had no forearms or hands), and many of the other impairments connected to this syndrome, such as severe speech and motor delays, poor social interaction and a tendency toward aspiration and chest infections (Kline et al. 2007). At 5 years old she was incontinent and not walking or talking. She was not participating in formal stimulation programmes. Brenda moved about the house by shuffling on her buttocks. She could sit independently and manipulate objects between her arms. She drank from a baby bottle without assistance. She could stand and give a few steps with minimal support around her hips. She made eye contact and communicated through gestures, facial expressions and noises.

Brenda’s grandmother was her primary caregiver. Her grandmother treated her like a baby and approached disability from a moral angle: ‘We cannot help it. It is God’s work’ (Grandmother, F, housewife, 56). Her mother lived elsewhere and only visited. They did not have contact with her father. They lived in a four-room government-supplied house. The house was clean and well kept, but had little furniture (Brenda and her grandmother shared a bed). They had indoor plumbing, but no electricity. Cooking was done on an open fire.

Brenda had potential to progress:

‘The way they [*doctors at the tertiary hospital*] explained it to me, she will get better, but not in the same time as a normal child. It will take a long time.’ (Mother, F, domestic worker, 21)

Progress would require rehabilitation:

‘The doctor said that the people who gave the exercises must come often.’ (Grandmother, F, housewife, 56)

She apparently received very little rehabilitation:

‘The people, who give her exercises, were here very long ago.’ (Grandmother, F, housewife, 56)

‘They came to the clinic once. I saw them once and gave a follow-up date, but I never saw them again … I planned to do early intervention, like the beginning of language. The continuity is lacking. You want to follow up very much, but you cannot always go on a home visit, since there are three and everyone must be somewhere [*they have one vehicle for their use*].’ (Therapist 1, F, tertiary education, 25)

A CHCC professional nurse said that Brenda did not receive rehabilitation because her condition was genetic. Brenda was not provided with any assistive devices, although a wheelchair was mentioned by primary- and tertiary-level healthcare service providers:

‘Sister [*name of professional nurse*] told me when the people come again I have to talk to them about a push chair for her.’ (Grandmother, F, no formal education, housewife, 56)

‘… the doctor in Kimberley said I must ask them for a push chair when they come again. But when will they come again that I can ask?’ (Grandmother, F, no formal education, housewife, 56)

No consumables for incontinence management were provided:

‘They do not give nappies. We buy nappies at [*name of shop*].’ (Mother, F, domestic worker, 21)

According to the grandmother, she received no training or education on the prevention of complications like aspiration or to improve function such as continence training, communication, mobility and self-care:

‘I … do not know how to do it [*exercises for Brenda*].’ (Grandmother, F, housewife, 56)

The grandma could not afford toys, furniture such as a table and chairs or appliances or devices such as a wheelchair.

### Carel

Carel^2^, 47 years old at the time of the study, was employed as a farm labourer when he contracted what sounds like an infectious disease of the spinal cord or brain several years ago. None of the persons interviewed could indicate when he contracted this disease or exactly what it was: ‘The doctor said he had spinal cord fever’ (Mother, F, no formal education, housewife, 81). It seems that he received extensive medical care at the time of the disease to the extent of being referred to a tertiary hospital and having his life saved from what, according to his sister, is usually a fatal condition. The disease left him permanently impaired, since his lower limbs were paralysed.

Carel lived with his mother, who cared for him. He was illiterate and received a disability grant. The house had two bedrooms, a bathroom with a flush toilet, a kitchen and a sitting room. Poverty was evident in the sparse, dilapidated furniture and worn floor coverings. Carel and his mother shared a bedroom. The house had two outside doors, each with four steep steps to ground level.

Previously, Carel used a wheelchair. When this wheelchair broke, it was not repaired or replaced for more than a year by the Department of Health of the provincial government of the Northern Cape Province. Without any means of mobility, Carel lay in bed and developed contractures. In 2010, a new wheelchair was delivered to their house by a therapist. The therapist did not assess him at this time and did not determine whether he could still transfer to and balance in the wheelchair. At the time of the study he could not transfer into, sit in or propel the wheelchair:

Mother: ‘I think it’s hopeless [*getting into the wheelchair*]. I have nobody to help me and he is very heavy as well.’ (Mother, F, housewife, 81)

Researcher: ‘And you cannot get into the wheelchair by yourself?’

Carel: ‘No.’ (M, no formal education, 47)

Researcher: ‘Did they show you how to get in the wheelchair?’

Carel: ‘No.’ (M, no formal education, 47)

Carel could eat by himself, but could not sit up, wash, dress or get out of bed. According to him and his family, he has no role in the household: ‘I do nothing but lie here’ (Carel, M, no formal education, unemployed, 47). It seems as if further interventions were considered at a later stage but not implemented:

‘He did go there [*referral hospital*]. They wanted to straighten his legs [*he had contractures of the knees and hips*], afterwards they said no. Then they wanted to amputate his legs. But we did not want that. It will be too painful.’ (Mother, F, housewife, 81)

The contractures in his legs decreased his functional abilities, such as getting into the wheelchair and using a bed pan:

‘He asked for the pan and I gave it to him and the bed stayed clean [*in the past*]. He used it for years, but now he cannot get onto it anymore. Because of the legs. They are stiff [*contractures*]. Now he cannot lift the legs to get onto the pan. I use vest rags. Like a baby.’ (Mother, F, housewife, 81)

Neither he nor his mother knew whether he was taught measures to prevent contractures. Previously he received a monthly supply of incontinence diapers:

‘We used to get it [*diapers*], but strangely it just stopped bluntly with no explanation. They just said it was finished. I did not ask why.’ (Mother, F, housewife, 81)

Home-based care had been rendered but was also stopped:

‘Two of them came to wash him in the mornings. Once a week on a Tuesday. Now they do not come anymore.’ (Mother, F, housewife, 81)

Healthcare service providers told him they could not assist him:

‘Last year. He went to the sister [*professional nurse*]. That time they said they cannot do anything to help him…. Well, now we do not go anymore.’ (Mother, F, housewife, 81)

Carel and his mother can recall outreach visits from therapists, but they could not describe what the therapists did. None of the therapists currently doing outreach in the community knew him.

### Additional information from therapists

According to the therapists, not all users who could benefit from their services were referred to them:

‘We struggle a lot with referrals.’ (Therapist 2, F, tertiary education, 23)

Late referrals negatively impact users’ prognosis:

‘The longer you wait, the more difficult it is to restore function.’ (Therapist 2, F, tertiary education, 23)

The focus of therapeutic interventions was on function:

‘To get them as functional as possible. We focus on what they struggle with in the house and how they can do things.’ (Therapist 3, F, tertiary education, 23)

The therapists found some users complained and others not:

‘Some patients come back [*to the clinic for therapy*] and others just disappear.’ (Therapist 2, F, tertiary education, 23)

Therapists could not provide explanations for what they saw as non-compliance.

According to the therapists, the provision of walking devices and hearing aids was adequate, but not that of wheelchairs:

‘You measure the person and send the application form to [*name of provincial hospital*]. Then you wait and see when you get it. They decide whose need is the biggest and who gets first. You can indicate on the form if the person will develop contractures or any other detrimental things that can happen.’ (Therapist 2, F, tertiary education, 23)

The therapists were aware of the limited time they spent in the community and how that impacted their service delivery negatively:

‘We lose a lot of time on the road. Four to five hours of the working day is spent driving. The time here is limited.’ (Therapist 2, F, tertiary education, 23)

They described a lack of communication between service providers and challenges with regard to the referral system:

‘A woman phoned this morning to say she phoned the clinic months ago to ask for an appointment with one of us, but nobody comes to see her. We have never heard of this woman. Thus somebody takes messages, but the messages do not reach us.’ (Therapist 3, F, tertiary education, 23)

‘No, we do not get answers back [*from the referral hospital*]. We write letters, but the referral back is very poor. The patient cannot give feedback to you. Then you try to phone. But you cannot phone with the folder number only. Every department has a different number for the patient.’ (Therapist 1, F, tertiary education, 25)

Therapists could not provide examples of CBR programmes or projects in the study community and could not theorise on how it might be feasible. They were unsure of the role home-based carers could play in the provision of rehabilitation services to the community and were not aware of the presence of home-based carers in the community:

‘I do not know if they have a role. I do not know if there are any.’ (Therapist 3, F, tertiary education, 23)

The first author had to explain the concept of intersectoral collaboration to the therapists, and they could not think of any examples.

## Discussion

**FIGURE 1:**
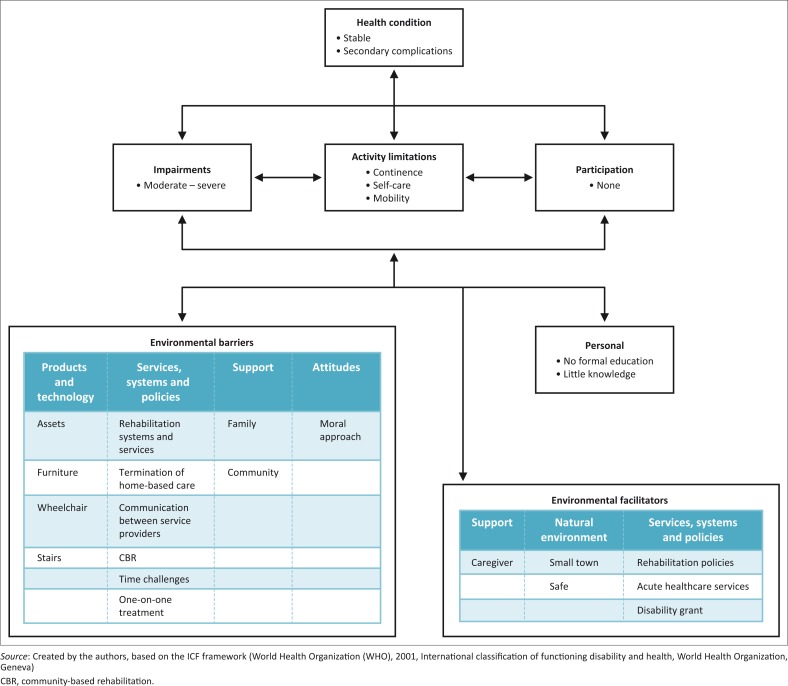
Interaction between domains according to the International Classification of Functioning, Disability and Health.

Both participants faced multiple challenges that in combination hampered participation and community integration. The effect of moderate to severe impairments on activities and participation was aggravated through personal and environmental barriers. Rehabilitation intervention or lack thereof failed to modify impairments and the environment, or compensate for loss of function, sufficiently to ensure community integration. The interaction between the various challenges and their impact on rehabilitation is presented in Figure 1 and will be discussed in an integrated manner.

Whereas rehabilitation serves as a focus for the discussion, rehabilitation service provision is in itself a barrier or facilitator to participation and is classified under the environmental domain of services, systems and polices. Policies and other guiding documents on the provision of rehabilitation in South Africa are in existence (DoH 2000, 2013) and are thus shown as a facilitator in Figure 1. These documents focus on the availability of high quality rehabilitation services. While direction is given on what type of services should be available at which level of care and the philosophies to be followed, these documents are largely silent about dealing with the practicalities of implementation. Strategic plans mostly focus on desktop paperwork and the development of committees (DoH 2013).

Findings from the case studies and the literature (Bateman 2012; Maart & Jelsma 2013; Ntamo et al. 2013) showed that implementation of these policies is challenging and that rehabilitation services and systems created barriers to rehabilitation service delivery. In the current study, service and system barriers are evident in the challenges with referral and communication, the limited therapy hours in the community, the lack of CBR and the challenges regarding provision of assistive devices, as shown in Figure 1.

The little time therapists spent in this community resulted in very limited therapy, especially since the preferred mode of therapy was one-on-one treatment. Based on the case study findings and challenges to rehabilitation in South Africa as presented in the introduction and findings by Puckree and Uthum (2013), we hypothesise that traditional one-on-one treatment sessions by therapists might not be the way to ensure ‘rehabilitation for all’ (DoH 2000) in the study setting and similar settings in South Africa. Transdisciplinary or interprofessional teamwork provides an alternative option, as does CBR.

Through transdisciplinary teamwork, integrated patient- and family-centred services can be provided in the face of complex needs. Benefits include cost-effectiveness, less intrusion in family units, holistic service delivery and greater opportunities for service deliverers to enhance their knowledge and skills. Skills are shared and interventions provided across professional boundaries (King et al. 2009; Sims, Hewitt & Harris 2015). In the study setting, transdisciplinary teamwork provided at clients’ homes might have led to more focussed interventions. Therapists can consult with colleagues (the 4 hours during which they drive to and from the town provides an opportunity for this) and experts and then provide interventions across professional boundaries (Bruder 2010). Modern technology can assist with sourcing information on rare conditions and resources. Lack of transport should not create an obstacle in a town that is no bigger than 4 km^2^ and has little violence. With some planning, the car can be parked at a central spot from where therapists should be able to walk the short distances to the houses of various clients. The large overlap in scope of practice will allow for critical basic rehabilitation intervention, caregiver training and transfer of skills long before scope of practice challenges come into play. In addition, we agree with Gaede and Versteeg (2011) that scope of practice should be expanded in rural areas where limited providers are available. Although this option will not solve the time dilemma, it might help to ensure that more people receive intervention on a given day and that the interventions are more focussed than therapy provided at the CHCC.

Transdisciplinary teamwork can be aided by peer mentors and CBR programmes. Neither participant was seen or treated as a valuable member of the households or communities. Negative thoughts and lack of expectations could have led to the creation of images of uselessness and helplessness (Harrison et al. 2010). Peer exposure to people with similar impairments that are integrated into their communities provides a way of dispelling these notions (Ljungberg et al. 2011; Haas et al. 2013).

South African policy advocates rehabilitation service delivery according to CBR principles (DoH 2000). No example of the implementation of any of the WHO health guidelines (WHO 2010) for CBR programmes could be identified in the setting. The case studies showed that rehabilitation needs were not identified and that referral, follow-up, early intervention and linkage with self-help or peer support and training groups were lacking. The focus group with therapists confirmed a lack of early referral, intervention and follow-up as well as the absence of CBR programmes. This absence of CBR is unfortunate because others have shown that CBR can have a positive impact on self-care activities and participation (Chappell & Johannsmeier 2009), aspects that both Brenda and Carel lacked.

However, some of the barriers identified might challenge the implementation of CBR in this community. The therapists spent too little time in the community to perform a situational analysis or to plan, design, implement and monitor a CBR programme. Thus, the initiative will have to come from persons with disabilities, the community or the professional nurses at the clinic. The moral approach to disability, poverty and lack of education might leave the persons with disabilities and the community with few resources and little will to implement CBR. Professional nurses throughout South Africa are overworked and pressed for time. The study findings also indicate that professional nurses working in this community might have insufficient understanding and knowledge of the role of rehabilitation, a lack that will negatively impact their understanding of the need for CBR and ability to initiate such programmes.

Due to the impact of the impairments on mobility, both participants needed wheelchairs and wheelchair services. Brenda never received a wheelchair; Carel had a wheelchair that broke. A lack of assets meant that his only means of replacement was through the Department of Health. Replacement through this source took more than a year. In this time he developed contractures and lost his ability to use a wheelchair. When his wheelchair was replaced, service providers did not realise that he could not use it anymore. Thus, an expensive resource stood unused. These findings confirm previous findings that wheelchair service provision to this community was challenged through a number of issues including referral, assessment, prescription, training and follow-up as described by Visagie, Scheffler and Schneider (2013).

Although the primary condition of both participants seemed to be stable, both showed evidence of secondary complications, which negatively impacted their activities and participation. This finding points toward a failure to successfully include education and training in rehabilitation service delivery. This perceived failure might be due to service-related factors such as the limited time therapists spent with participants, the described poor communication between service providers and a lack of knowledge amongst the professional nurses.

However, personal and environmental contextual factors probably added to the challenge of implementing prevention strategies. Whereas the commitment shown by the primary caregivers served as a facilitator, both carried the burden of care to a large extent on their own. They were also illiterate, approached disability from a moral angle and were of ages where their own physical powers were declining. In combination, these factors might have decreased their understanding of and ability to perform activities to prevent secondary complications. In the case of Brenda, the smoke from cooking on an open fire due to not being able to afford a stove could have aggravated her tendency to chest infections. Support from family, friends, community members and formal services such as home-based care might have assisted.

The findings show that home-based care services were provided in the community, but home-based carers mainly provided directly observed therapy at the time of the study, and therapists were not aware of their presence in the community. This is a cadre of healthcare service providers that can be developed into community health workers. According to the South African National Development Plan 2030 (RSA 2012), they are an important component of primary healthcare outreach teams. They should provide community-based healthcare services that include rehabilitation (DoH 2013). To do this, they must be trained and supported by therapists.

## Conclusion

It seems as if only the impairments of the case study participants were visible, whereas none of their potential was realised. Neither case study participant was integrated into the community; both experienced higher levels of disability than one would expect from their impairments. Rehabilitation service delivery was challenged through service, environmental and personal barriers. These two cases probably represent the lack of rehabilitation in its most extreme, but the setting and cases are not unique in South Africa. The situation in this community might be no different from many other communities where the need for rehabilitation greatly exceeds the availability of services with a resultant negative impact on user outcomes.

### Recommendations

A move away from individual therapy toward transdisciplinary teamwork supported by family members, community health workers, peer mentors and CBR programmes is recommended in remote rural settings. It is recommended that the implementation of these strategies be done first on a pilot basis, with careful monitoring to determine feasibility and effectiveness. Therapists are ideally situated to explore the feasibility of such programmes and to start implementing them in various communities. There is a need to explore why CBR programmes are not implemented in the setting and whether it is implemented in similar settings. The extent to which pre-graduate training of various professional groups addresses transdisciplinary teamwork and the ability of junior therapists to function in transdisciplinary teams must also be explored.
